# Neonatal face-to-face interactions promote later social behaviour in infant rhesus monkeys

**DOI:** 10.1038/ncomms11940

**Published:** 2016-06-14

**Authors:** Amanda M. Dettmer, Stefano S. K. Kaburu, Elizabeth A. Simpson, Annika Paukner, Valentina Sclafani, Kristen L. Byers, Ashley M. Murphy, Michelle Miller, Neal Marquez, Grace M. Miller, Stephen J. Suomi, Pier F. Ferrari

**Affiliations:** 1 Laboratory of Comparative Ethology, Eunice Kennedy Shriver National Institute of Child Health and Human Development, National Institutes of Health, Poolesville, Maryland 20837, USA; 2 Department of Population Health and Reproduction, University of California, Davis, California 95616, USA; 3 Department of Psychology, University of Miami, Coral Gables, Florida 33146, USA; 4 Winnicott Research Unit, University of Reading, Reading RG 6AL, UK; 5 Johns Hopkins Bloomberg School of Public Health, Baltimore, Maryland 21205, USA; 6 Institute for Health Metrics and Evaluation, University of Washington, Seattle, Washington 98121, USA; 7 Clinical and School Psychology, University of Virginia, Charlottesville, Virginia 22904, USA; 8 Department of Neuroscience, University of Parma, 43125 Parma, Italy

## Abstract

In primates, including humans, mothers engage in face-to-face interactions with their infants, with frequencies varying both within and across species. However, the impact of this variation in face-to-face interactions on infant social development is unclear. Here we report that infant monkeys (*Macaca mulatta*) who engaged in more neonatal face-to-face interactions with mothers have increased social interactions at 2 and 5 months. In a controlled experiment, we show that this effect is not due to physical contact alone: monkeys randomly assigned to receive additional neonatal face-to-face interactions (mutual gaze and intermittent lip-smacking) with human caregivers display increased social interest at 2 months, compared with monkeys who received only additional handling. These studies suggest that face-to-face interactions from birth promote young primate social interest and competency.

## Abstract (web summary)

Like humans, neonatal primates engage in face-to-face interactions with their mothers from an early age. Dettmer and colleagues demonstrate that in monkeys, increasing neonatal face-to-face interactions enhances social interest in infants of two and five months.

## Graphical abstract

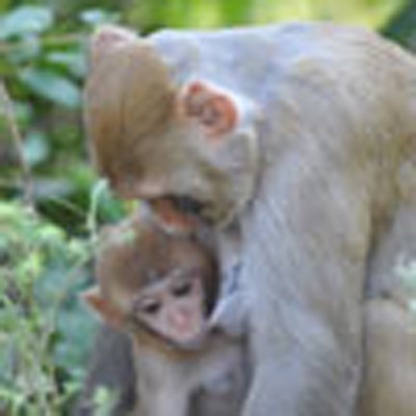

Highly social species, including human (*Homo sapiens*) and nonhuman primates (NHPs), evolved a variety of sociocognitive skills and behaviours—including complex facial expressions, grooming and play—that facilitate cooperation among group members^1^. In these societies, characterized by complex and extended social interactions, social competency is critical for survival and reproductive fitness^2^. However, the mechanisms by which individuals acquire social competence early in development are not well understood.

One mechanism proposed to support early social development is face-to-face interactions^3^,^4^,^5^. Face-to-face interactions between newborns and caregivers occur across many human cultures^6^,^7^, and have also been reported for some NHPs (for example, chimpanzees, *Pan troglodytes*^8^; Japanese macaques, *Macaca fuscata*^9^; rhesus macaques, *Macaca mulatta*^3^), especially during or following physical separation^3^,^5^,^8^,^10^. In NHPs, face-to-face interactions include mutual gazing (MG), which is often accompanied by facial gestures such as lip-smacking^3^. Similar to humans^11^,^12^, NHPs show considerable variability in face-to-face interactions with newborns^3^,^5^,^8^, and there is evidence that disruption of the mother–infant bond in rhesus monkeys negatively impacts infants' social and physiological development^13^. One possible mechanism for this disruption is the lack of face-to-face interactions between mother and infant. Primates, including humans, are attracted to the eye region of faces from the first weeks of life^14^,^15^, and prefer direct gazes to other visual stimuli^16^,^17^,^18^,^19^. In infant rhesus monkeys, increased visual attention to eyes is associated with other social skills such as neonatal imitation^15^. Face-to-face interactions offer opportunities for the infant to learn key information about the caregiver, about species-specific interactions, and about the foundations of early emotional communication^3^. However, what remains unclear is the extent to which sustained face-to-face interactions and their variability influence the expression of social behaviour later in development.

We addressed this question by studying the effects of neonatal MG on the later social behaviour of infant rhesus monkeys, a highly social Old World primate species with strong mother–infant bonds and social complexity in adulthood^20^. We predicted that, if MG is a primary driver of the development of social skills, then monkey infants who receive more MG should be more social later in development. To test this prediction, we first observed naturally-occurring variation in mother–infant face-to-face interactions (see Supplementary Movie 1) to determine whether the quantity of face-to-face interactions predicts infants' later developing social behaviour (Experiment 1). In this study, we focused on face-to-face interactions that occurred during close physical proximity because it was not possible to observe such interactions when monkeys were out of contact (see Methods section). We then carried out a second study in which infants were randomly assigned to receive varying levels of face-to-face interactions (Supplementary Movie 2) and physical contact with human caregivers in a controlled rearing environment (Experiment 2). Thus, through a combination of both naturalistic observations and experimental manipulations we were able to gain a more complete picture of the contributions of neonatal MG to the developing infants' social interest and engagement in social behaviours. We report here that infants experiencing more face-to-face interactions in the first month of life exhibit more social interest at 2 and 5 months, suggesting that face-to-face interactions from birth promote young primate social competency.

## Results

### Results from studies of semi free-ranging monkeys

**Figure 1:**
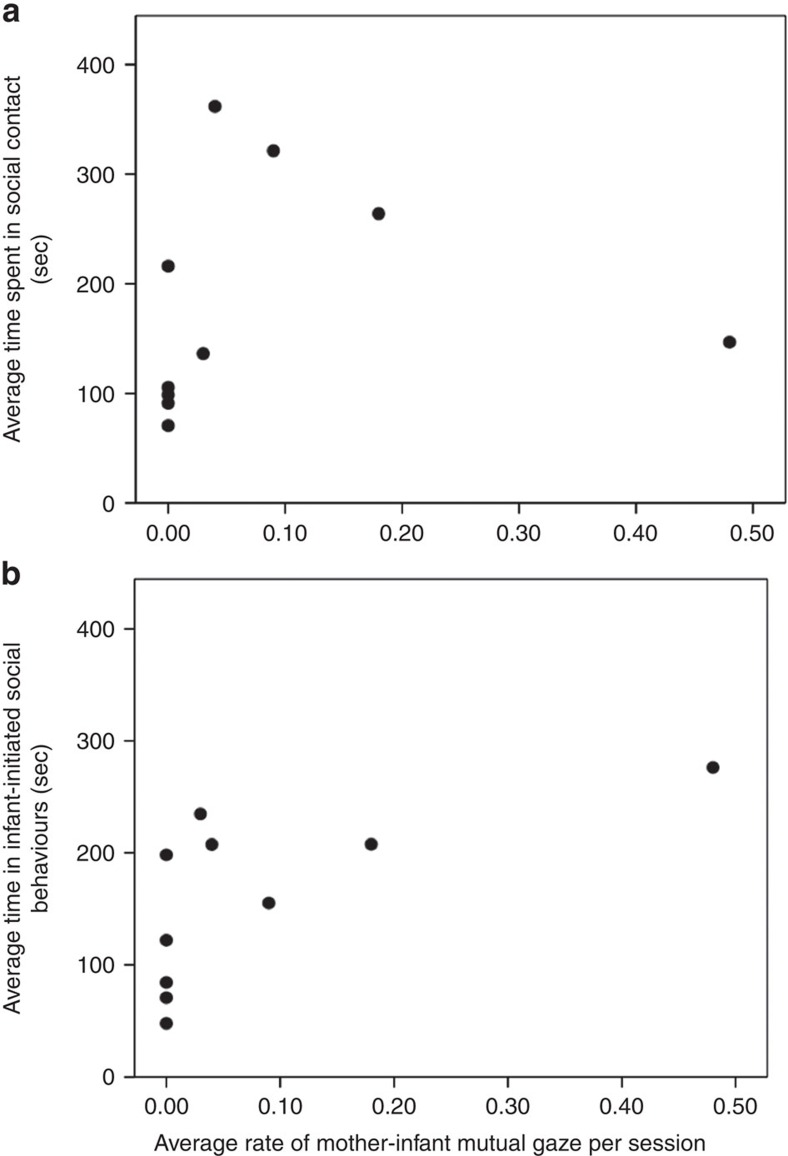
How mother–infant face-to-face interactions influence infant social behaviour. Results from Experiment 1 examining neonatal mother–infant face-to-face interactions and later infant social behaviour. (**a**) Correlation between rates of mother–infant mutual gazing in the first month of life and time the infant spent in social contact at month 2. Rate of gazing=total frequency of mutual gaze in first 30 days/total number of 15-min sessions in first 30 days. *N*=10. (**b**) Correlation between rates of mother–infant mutual gazing in the first month of life and time infants spent in social behaviours (for example, groom, play and social contact) they initiated. Rate of gazing=total frequency of mutual gaze in first 30 days/total number of 15-min sessions in first 30 days. *N*=10.

In Experiment 1, we studied MG in semi free-ranging rhesus monkey mothers and infants (*N*=10) living in a large, 2 ha enclosure. Researchers recorded the frequency of MG between mothers and infants across the first three months of life^5^, and tracked infants' social behaviour (that is, social play, close proximity to other monkeys and grooming) for the first 5 months of life (see Methods section). Infants who engaged in MG more frequently with their mothers in the first month of life were more sociable later on: they spent more time in social contact with other monkeys at 2 months of age (Spearman's correlation; *r*_(s)_=0.68; *P*=0.031; Fig. 1a), and they initiated more social interactions at 5 months of age (Spearman's correlation; *r*_(s)_=0.78; *P*=0.007; Fig. 1b). Infants did not preferentially initiate social behaviours with their mothers; instead they initiated social behaviours with all types of partners: other adults, other infants, juveniles and their mothers.

### Results from studies of monkeys in a controlled environment

In Experiment 2, infants were reared in a nursery by human caregivers and had continual contact with peers^21^. Mother–infant physical contact can promote social behaviours^22^,^23^, which may be driving the increased sociality observed in Experiment 1. We, therefore, carried out a second study to determine the extent to which increased physical contact or increased face-to-face interactions influenced later social behaviour. We randomly assigned infants to receive standard care (*N*=17), increased handling by human caregivers without face-to-face contact (caretakers' faces were covered; *N*=15), or both increased handling and face-to-face interactions (MG and intermittent lip-smacking; *N*=16), for the first 4 weeks of life^24^ (see Methods section). We then tracked infants' social development with two measures across the first 5 months of life: (1) by assessing infants' preference for a social (that is, a macaque mother with her infant being groomed by another adult) versus nonsocial (that is, geometric shape) videos using an eye tracker, and (2) by measuring social behaviour during infants' daily interactions with same-aged peers.

**Figure 2:**
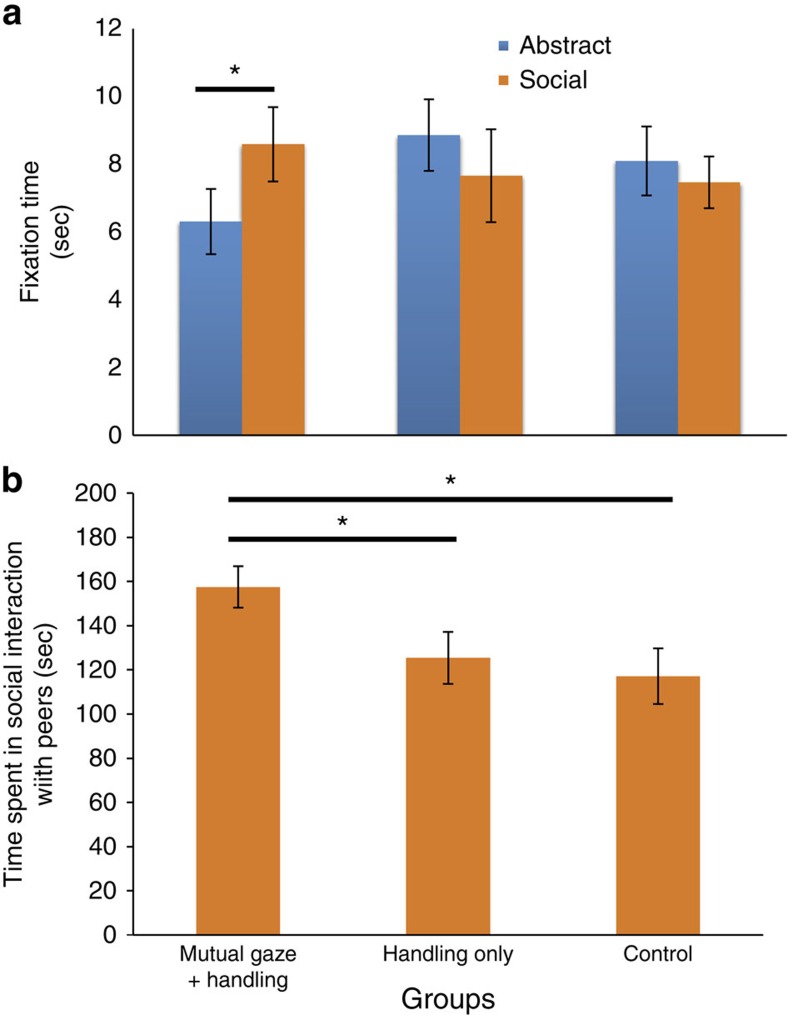
Face-to-face interactions, but not extra handling, influence social development. Results from Experiment 2: (**a**) Effect of face-to-face+handling treatment on average time looking at social (orange) versus nonsocial/abstract (blue) stimuli during the eyetracking task at 2 months, and (**b**) effect of face-to-face+handling treatment on average time spent in social contact with peers at two months. Face-to-face+handling, *N*=16; Handling, *N*=15; Standard care, *N*=17. *P<0.05. Error bars reflect s.e.m.

Infants who experienced additional face-to-face interactions spent more time at 2 months of age looking at the social stimulus than the nonsocial stimulus (paired sample *t*-test; *t*_(15)_=2.38; *P*=0.031, *d*=0.55, Fig. 2a), whereas infants who experienced handling-only or standard care exhibited no preference (paired sample *t*-test; handling-only: *t*_(14)_=0.837; *P*=0.416; standard care: *t*_(16)_=0.446; *P*=0.661, Fig. 2a). At this age, we also found a significant effect of face-to-face interaction on the amount of time infants spent in social interaction with peers (analysis of variance (ANOVA); *F*_(2,40)_=4.125; *P*=0.023, *η*^2^=0.141; Fig. 2b): infants in the face-to-face condition spent more time interacting with peers (mean±s.d.=157.5±36.4 s) than infants in the handling-only (mean±s.d.=125.4±45.4 s; *t*_(29)_=2.133; *P*=0.042, *d*=0.78; *post hoc t*-tests) or standard care groups (mean±s.d.=117.1 s; *t*_(31)_=2.567, *P*=0.016; *d*=0.92; *post hoc t*-tests). There was, however, no difference in social behaviour between the handling-only and standard care groups (*t*_(29)_=0.48; *P*=0.635; *post hoc t*-tests). Although surrogate peer-reared infants engaged in social interactions for significantly longer (153.3±38.1 s) than peer-reared infants (ANOVA; 117.3 s±47.7 s; *F*_(1,40)_=7.956, *P*=0.007, *η*^2^=0.136), there was no significant effect of the interaction between treatment group (face-to-face+handling, handling-only, standard-reared) and nursery rearing condition (peer-reared, surrogate peer-reared; ANOVA; *F*_(2,40)_=1.138, *P*=0.331). These findings indicate that the effect of the stimulation on infant social behaviour is not driven by any specific rearing condition. Finally, it is possible that the infants in the face-to-face condition were more likely to seek social contact with their peers because they experienced higher levels of anxiety. However, we did not find any effect of the face-to-face condition on rates of self-scratching (ANOVA; *F*_(2,40)_=0.361, *P*=0.699), time spent in ventral clinging on peers (ANOVA; *F*_(2,40)_=2.170, *P*=0.127) or time in contact with the surrogate (ANOVA; *F*_(2,40)_=1.080, *P*=0.349), suggesting that the effect of face-to-face interaction on social behaviour is not due to the infants seeking reassurance from their peers or to a generalized reduction of anxiety. No group differences were observed on either measure at 5 months of age.

## Discussion

Our combined observational and experimental studies demonstrate that, in both a naturalistic and a laboratory setting, early face-to-face interactions between newborn primates and their caregivers significantly affect infants' social behaviour later in development: monkeys engaging in more face-to-face interactions as newborns spend more time in social contact with conspecifics, look longer at social stimuli, and initiate more social interactions. These effects do not appear to be due to increased physical contact between the newborn and caregiver, but appear to be driven by face-to-face interactions.

Interestingly, it has been reported that the frequency of mother–infant mutual gaze not involving lip-smacking predicted the amount of lip-smacking that the infants received from their mothers^3^. Moreover, lip-smacking by adults to infants coincides with mutual gaze^3^, suggesting that in order for lip-smacking to occur, mutual gaze must be occurring. While these events are difficult to record in the field, as they require close proximity and detailed video microanalysis, this and other studies suggest they are more common in nonhuman primates than previously thought^3^,^5^, and they probably have a significant impact on infants' affective and cognitive development, as also proposed in humans^25^,^26^. Further studies are needed to assess which specific component of mother–infant face-to-face interactions play a crucial role in the development of infant macaques social skills.

All infants, including those who are not exposed to a high rate of lip-smacking from their own mothers, are likely to experience these mutual interactions with other individuals of the social group, which may explain why infants who were not observed to receive face-to-face interactions with their mothers do not show gross social dysfunctions. This seems particularly true after the first month of life, when such mutual exchanges between macaque mothers and infants dramatically decrease^3^. Indeed, typically reared infant rhesus macaques become more independent after their first month of life, when their interest in and proximity to same-age peers and other individuals within the troop steadily increase^27^. By 6 months of age, infants typically spend the majority of their daytime hours away from their mothers and engaged with peers in social interactions^27^.

Previous work demonstrated that infant monkeys randomly assigned to receive more face-to-face interactions (mutual gaze and lip-smacking) were more likely to imitate facial gestures at one week of age, compared to infants who did not receive these additional interactions^24^. The present findings suggest that these face-to-face interactions may have even longer-lasting effects on infant social behaviour beyond the newborn period. However, the controlled experiment revealed no significant effects of the face-to-face interaction on social behaviour with peers beyond the second month of life, while the field experiment showed effects at five months. This discrepancy is likely due to the fact that in the field face-to-face interactions between mothers and infants continue well beyond the first month of life^5^. In contrast, in the controlled setting, the intervention only lasted for the first month of life. It is likely that continuing the face-to-face interactions would have resulted in longer-lasting social effects in the nursery group as well.

There was considerable natural variability in the extent to which mothers in the field interacted with their infants: only about half of the mothers were observed engaging in these face-to-face interactions, consistent with previous reports^3^,^5^,^8^. Those infants whose mothers were not observed to engage in face-to-face interactions with them nonetheless went on to develop normally. That is, there did not seem to be any obvious dysfunction in these mother-raised infants as a function of not having high rates of mutual gaze with their mother as neonates, at least during the first 5 months of life. Furthermore, because we did not observe such interactions it does not mean they did not occur; interactions may have occurred at times in which they were not observed or may have been subtle or difficult to detect in this context. Nonetheless, these findings suggest that for some dyads, such mother–neonate interactions may be quite rare, and perhaps there may be other causes of variability in these interactions besides human interference (for example, maternal experience and infant sex^5^).

It is not yet clear what the mechanisms are underlying the differential social behaviour for infants receiving variable early caregiving. One possibility is that variability in face-to-face interactions may modulate the activation of the oxytocin system^28^, as oxytocin is a neuropeptide that plays a key role in mother–infant bonding and promotes affiliative relationships^29^ and that may influence downstream social development^30^. Recent work has shown that endogenous oxytocin in children can be increased through parental contact^29^ and that exogenous oxytocin increases eye contact in humans with and without autism^31^. It is, therefore, possible that different levels of oxytocin in infants and/or in caregivers influence the frequency of face-to-face interactions, or *vice versa*, which might ultimately promote differential levels of social engagement^29^. In support of this, aerosolized oxytocin increases affiliative behaviour in newborn macaques, especially among infants with stronger social skills, suggesting oxytocin may amplify infants' intrinsic social interest^30^.

Our data suggest that the development of sophisticated social interactions and complex social systems might have been an important factor driving the evolution of mother–infant social gazing. Individuals living in a stable social group need to employ advanced social skills both to coordinate their own behaviours with the behaviours of other group members, and to solve direct and indirect conflicts that originate from competition over resources^32^. The primate species in which mutual gazing has been reported to date, namely macaques (for example, rhesus macaques^3^; Japanese macaques^9^), geladas (*Thereopithecus gelada*^33^), chimpanzees^8^ and humans^10^,^11^, are all highly social species characterized by multi-male multi-female social systems. Interestingly, in these species, individuals use social tactics to secure access to resources and increase their reproductive success^34^,^35^. We suggest that, in these species, the acquisition of social skills starts in infancy, since being able to learn these skills from caregivers through, for instance, face-to-face interactions promotes social competence, which is critical for survival in adulthood in complex societies^32^.

## Methods

### Ethical approval

All procedures were approved by the NICHD Animal Care and Use Committee.

### Experiment 1

Rhesus monkey mother–infant dyads (*N*=10; 4 male infants) were born and raised at the Laboratory of Comparative Ethology's five-acre field station and the NIH Animal Center in Poolesville, Maryland. We studied dyads in the birth seasons (spring and summer) of 2013 and 2014. This semi free-ranging population of ∼80 monkeys has been well characterized^5^,^36^. Mothers and infants were undisturbed for the duration of the study.

Three observers recorded mother–infant interactions, trained to >85% reliability according to the methods detailed by Ferrari *et al*^3^,^5^. We conducted live focal animal observations^5^,^37^ between 09:00 and 17:00 hours, one to two times per day, 5 days per week for the first 30 days of the infant's life; three times per week during days 31–60; and once per week during days 61–90. We coded dyads for 15 min, and sessions were coded from the infant's perspective. We discarded sessions if the focal animals moved out of sight or if either the mother or infant fell asleep for over 50% of the session^5^. We recorded the frequencies of mutual gazing, defined as eye-to-eye contact between mother and infant lasting at least 3 s (Supplementary Movie 1), in each 15-min session.

We observed infants from days 30–180 for all occurrences of behaviours using focal animal observations^5^,^37^. From days 30–60, infants were coded twice per week for 20 min each session; from day 60 onwards infants were coded weekly for 30 min each session. We recorded behaviours on a MobileDemand xTablet T7200 (Hiawatha, Iowa, USA) using JWatcher software^38^. For this study, the following behaviours were coded as initiated or received by the infant:

1. Social play: play face, non-aggressive chasing, tagging, swatting, bobbing, biting, pulling, lunging, mouthing or wrestling (rough and tumble) directed towards another animal.
2. Social contact: in physical contact or within arm's reach of another animal.
3. Social grooming: cleaning/picking/stroking hair.

We calculated the average rates of mutual gazing between mother and infant for the first month of life. We calculated average durations and frequencies of each of the social behaviours for each month between 2–6 months. We used Spearman's correlations to relate mutual gazing with durations of each of the behaviours at each month of age. In addition, we calculated a composite ‘initiate social' score for each month (that is, from 2 to 6 months) by averaging the durations of all social behaviour (social play+social contact+grooming) that the infant initiated. We again used Spearman's correlations to relate mutual gazing with the initiation of social behaviours at each month of age. We ran these latter correlations for interactions between infants and all other social partners, and for interactions between infants and separate classes of social partners (that is, mother, adult female, adult male, juvenile and other infant).

### Experiment 2

Infant rhesus macaques (*N*=48; 27 males) were raised in a nursery from the day of birth following established procedures in our laboratory^30^,^39^,^40^. For unrelated projects, some infants (*N*=28) were reared in groups of four (peer-reared), while others (*N*=20) were reared in single cages outfitted with cloth-covered surrogates and given daily 2-h play sessions (surrogate-peer-reared), beginning at ∼40 days of age. Before this time, infants were housed in an incubator for the first 14 days of life, then transferred to a single cage until group formation. The single cages were all contained in the same room so that infants had constant visual and auditory contact with one another.

On the day of birth, we randomly assigned infants to one of the three conditions. In two of these conditions infants received additional daily stimulation: a face-to-face+handling condition (FF, *N*=16) and a handling-only (HDL, *N*=15) condition. We compared these stimulated infants with a standard-reared control group (SR, *N*=17), who received no additional social interactions beyond standard rearing^39^,^40^. Each stimulation session was carried out by one of approximately a dozen different caregivers, so that infants did not form an attachment to any one experimenter.

In the FF condition, a human caregiver attempted to engage the infant in mutual gaze and, on doing so, directed lip-smacking gestures (LPS) at the infant for ∼5 s, followed by a 10-second neutral still-face period (Supplementary Movie 2). This LPS-still period was repeated every 30 s, for a total face-to-face interaction lasting 5 min per session. We chose LPS because it is a common, affiliative behaviour mother rhesus macaques direct to their infants during face-to-face interactions^3^ and infants imitate LPS in the first week of life^40^. In the HDL condition, a human caregiver held the infants for the same duration (5 min), but wore a face cover to prevent the infants from seeing the caregiver's face. For the first 2 weeks of life, we administered both FF and HDL four times per day (at ∼10:00, ∼12:00, ∼14:00 and ∼18:00 hours) during weekdays and twice per day on the weekend (at ∼10:00 and ∼12:00 hours). In the third week of life, we administered FF and HDL three times per day (at ∼10:00, ∼12:00 and ∼14:00 hours) and once per day on weekends (at ∼10:00 hours), while in the fourth week of life we administered FF and HDL twice per day (at ∼10:00 and ∼12:00) during weekdays and once per day on weekends (at ∼10:00 hours). The purpose of this gradual reduction in sessions was to prevent infants from growing accustomed to regular stimulation that would end abruptly, and to mimic naturally-occurring declines in mother–infant face-to-face interactions across development^3^.

At 40–50 days of age (median=41 days), we tested infants in an eyetracking task to assess preference for social interactions. We recorded infants' eye movements via corneal reflection using a Tobii T60XL (*n*=38) or a Tobii TX300 (*n*=10) eye tracker and a sampling rate ≥60 Hz. We used Tobii Studio software (Tobii Technology, Sweden) to collect and summarize the data.

One experimenter held each infant *ca.* 65 cm in front of the screen, swaddled in a soft blanket. We calibrated each infant using a five-point calibration to Tobii Studio's pre-set locations. Infants viewed one 30-s video (Supplementary Movie 3) that depicted a social monkey scene on one side (a macaque mother with her infant being groomed by another adult) and a nonsocial scene on the other side (abstract shapes continuously moving across the screen). Location of the social scene was counterbalanced (left/right) between infants. We repeated the task when infants were 149–246 days (median=161 days); one infant was not re-tested at this older age for non-experimental reasons.

We observed infants in their social groups (that is, during play sessions in the case of peer-reared infants) twice per week, once in the morning and once in the afternoon, using 5-min focal animal sessions^37^. We recorded the following interactions:

1. Social contact: when the infant was either in physical contact or in close proximity (within arm's reach) of a peer.
2. Play: play behaviours that included play face, non-aggressive chasing, tagging, swatting, bobbing, biting, pulling, lunging, mouthing and wrestling (rough and tumble).
3. Social grooming: cleaning/picking/stroking hair.
4. Self-scratches: raking one's own hair or skin with fingernails including large movements of arm.
5. Ventral clinging: ventral contact with peers.
6. Surrogate: time spent inside the surrogate.

We collected data on self-scratching, ventral clinging and time spent in the surrogate, as these are considered reliable indicators of anxiety^41^.

For the eyetracking task, we drew areas of interest for each side of the screen. We extracted total fixation durations using the Tobii filter in Tobii Studio (velocity: 35 pixels per window; distance threshold: 35 pixels). We calculated a preference score for the social video (social /(social+nonsocial)) and compared the amount of time infants looked at the social versus the nonsocial stimuli using a paired sample *t*-test.

For social interactions, we created a composite social score by taking the average time infants spent in social contact, play and grooming. This social score, as well as mean rates of self-scratching, ventral clinging and time spent in the surrogate were calculated at two different time points: (1) at 2 months (that is, between 36 days, when infants were first introduced to the social group, and 60 days) and (2) at 5 months (that is, 121–150 days). We could not include two infants (one in the FF and one in the SR condition) at 2 months, because they were introduced to the social group when they were older than 2 months. For each time point, we ran one-way ANOVAs that included the behaviour of interest as dependent variable, with condition (FF, HDL and SR), rearing (peer-reared, surrogate-peer-reared) and their interaction as independent variables, and *post hoc t*-tests to conduct pair-wise comparisons.

### Data availability statement

The authors declare that the data supporting the findings of this study are available within the article's supplementary files (Supplementary Data 1 and 2).

## Additional information

**How to cite this article**: Dettmer, A. M. *et al.* Neonatal face-to-face interactions promote later social behaviour in infant rhesus monkeys. *Nat. Commun.* 7:11940 doi: 10.1038/ncomms11940 (2016).

## Supplementary Material

Supplementary Movie 1 Mutual gazing between a rhesus monkey mother and her infant in Experiment 1.

Supplementary Movie 2 The face-to-face + handling treatment provided in Experiment 2.

Supplementary Movie 3 One of the 30 s videos shown to rhesus monkey infants for the eyetracking assessment in Experiment 2.

Supplementary Data 1 Data for Experiment 1

Supplementary Data 2 Data for Experiment 2
